# ZBP1 senses *Brucella abortus* DNA triggering type I interferon signaling pathway and unfolded protein response activation

**DOI:** 10.3389/fimmu.2024.1511949

**Published:** 2025-01-09

**Authors:** Marco Túlio R. Gomes, Erika S. Guimarães, Sergio C. Oliveira

**Affiliations:** ^1^ Departamento de Bioquímica e Imunologia, Instituto de Ciências Biológicas, Universidade Federal de Minas Gerais, Belo Horizonte, Brazil; ^2^ Departamento de Genética, Ecologia e Evolução, Instituto de Ciências Biológicas, Universidade Federal de Minas Gerais, Belo Horizonte, Brazil; ^3^ Institut Pasteur de São Paulo, São Paulo, Brazil; ^4^ Departamento de Imunologia, Instituto de Ciências Biomédicas, Universidade de São Paulo, São Paulo, Brazil

**Keywords:** *Brucella abortus*, macrophage, type I interferon, upr, ZBP1

## Abstract

The innate immune system promptly detects and responds to invading pathogens, with a key role played by the recognition of bacterial-derived DNA through pattern recognition receptors. The Z-DNA binding protein 1 (ZBP1) functions as a DNA sensor inducing type I interferon (IFN) production, innate immune responses and also inflammatory cell death. ZBP1 interacts with cytosolic DNA via its DNA-binding domains, crucial for its activation. *Brucella abortus* is the etiologic agent of brucellosis in livestock and humans, leading to significant economic losses and public health impact. Despite other innate immune sensors that recognize *B. abortus* DNA, including Toll-like receptor 9 and the Stimulator of interferon genes (STING), here we evaluated the ZBP1 participation as a cytosolic receptor sensing *Brucella* infection. Using macrophages derived from ZBP1 knockout (KO) mice we demonstrated that ZBP1 partially contributes to *IFN-β* expression upon *B. abortus* infection or *Brucella* DNA transfection. The knockdown of STING by siRNA decreased the residual IFN-β signal elicited by *B. abortus* infection, demonstrating the presence of a redundant cytosolic DNA-sensing mechanism driving type I IFN production. Furthermore, ZBP1 is involved in type I IFN signaling inducing *IRF-1* expression. Additionally, ZBP1 also contributes to Unfolded Protein Response (UPR) activation during infection. However, ZBP1 does not influence the production of proinflammatory mediators, inflammasome activation and it is dispensable to control bacterial infection in mice or replication in macrophages. This study highlights the complex interactions of *Brucella* components with innate immune receptors and identifies ZBP1 as a sensor for *B. abortus* DNA-induced IFN-β response.

## 1 Introduction

The innate immune system depends on its ability to promptly recognize invading pathogenic microbes as foreign and then take action to eliminate the threat (1). In that context, the detection of bacterial-derived DNA is central to mount an effective immune response against diverse pathogens (2). The Z-DNA binding protein 1 (ZBP1), also named DAI (DNA-dependent activator of interferon-regulatory factors), was identified as a DNA sensor inducing type I interferon (IFN) production and innate immune responses (3). Mechanistically, ZBP1 binds to cytosolic DNA through interactions involving its DNA-binding domains, which are required for its full activation (4). Subsequently, ZBP1 drives the activation of interferon regulatory factor 3 (IRF3), promoting the transcription of type I IFN (3). Similarly, activation of the cyclic GMP-AMP synthase (cGAS)-stimulator of interferon genes (STING) pathway also induces type I IFN production via IRF3 (5). This underscores the crosstalk between ZBP1 and cGAS-STING signaling pathways (6). In addition to type I IFN production, the induction of inflammatory cell death known as PANoptosis has also been attributed to ZBP1 activation (7). Activation of ZBP1 enables interaction with receptor-interacting serine/threonine-protein kinase 1 (RIPK1) and 3 (RIPK3), which can promote pyroptosis, necroptosis, and apoptosis (PANoptosis) by activating components such as NLRP3 inflammasome, mixed lineage kinase domain-like protein (MLKL), and caspase-8 (7–9). Several microorganisms are detected by ZBP1 through recognition of pathogen-derived nucleic acids, triggering the transcription of IFNs or the initiation of PANoptosis (6).For instance, ZBP1 activation by murine cytomegalovirus (MCMV) restricts viral replication by promoting host cell death (10). In addition, ZBP1 detects influenza A virus (IAV), triggering cell death and inflammation associated with IAV-related mortality (11). In contrast to the well-established role of ZBP1 as a viral sensor, its function during bacterial infection is less understood. Although, ZBP1-dependent cell death has been reported in infections with *Francisella novicida* (12) and *Mycobacterium tuberculosis* (Mtb) (13).

The facultative intracellular Gram-negative bacterium *Brucella abortus* is the causative agent of the global zoonotic disease brucellosis (14). In livestock, *B. abortus* promotes abortion and infertility, resulting in significant economic losses; in humans, brucellosis can potentially cause undulant fever, endocarditis, arthritis, and meningitis (15, 16). Brucellosis represents a major public health concern, and treatment is often challenging, requiring prolonged courses of multiple antibiotics (17). Thus, understanding the complex host mechanisms that recognize components of *Brucella* is crucial for developing effective treatments for brucellosis.

Over the past years, several receptors have been characterized as innate immune sensors for components of *B. abortus*, particularly host receptors that recognize pathogen-derived nucleic acids (18). For instance, *B. abortus*-derived DNA activates Toll-like receptor 9 (TLR9) through sensing of unmethylated CpG motifs (19). Moreover, the inflammasome receptor Absent in melanoma 2 (AIM2) senses cytosolic *B. abortus* DNA, promoting the activation of caspase-1 and secretion of IL-1β (20). *B. abortus*-derived DNA also activates the STING pathway, which induces the production of type I IFN, leading to an Interferon regulatory factor-1 (IRF-1)-dependent signaling cascade (21). In addition, STING activated by *B. abortus* infection triggers the Unfolded Protein Response (UPR), which is a conserved stress response in the endoplasmic reticulum (ER) initiated by the accumulation of misfolded proteins (22). The common downstream targets of the UPR pathway, such as binding immunoglobulin protein (BiP) and spliced X-box binding protein 1 (XBP1), are detected upon *B. abortus* infection through a STING-dependent mechanism, which is linked to STING-dependent IFN-β production (22). Given this complex interaction of *Brucella* components with a variety of innate immune receptors, we aimed to determine the participation of ZBP1 during *Brucella* infection. Here, we demonstrated that ZBP1 acts as a *B. abortus* DNA receptor driving IFN-β expression. Moreover, IRF-1 signaling and the UPR response are partially dependent on ZBP1 activation, although ZBP1 is not essential for controlling *B. abortus* infection.

## 2 Materials and methods

### 2.1 Animals

Wild-type (WT) C57BL/6 mice were obtained from the Federal University of Minas Gerais (UFMG) animal facility. ZBP1 knockout (KO) mice were provided by Prof Shizuo Akira from Osaka University (Japan). STING KO mice were described earlier (23). All mice were housed in a pathogen-free laboratory facility. Male and female mice aged 8-12 weeks were utilized for the study. All experimental protocols were reviewed and approved by the Animal Studies Committee (protocol CEUA/UFMG 69/2020).

### 2.2 Bacterial strains and growth conditions

*Brucella abortus* strain 2308 was acquired from our laboratory collection. The bacterium was cultured in Brucella broth (BB) medium (BD Pharmingen, San Diego, CA) for 3 days at 37°C under constant agitation before use. The optical density (OD) of the culture was measured at 600 nm using a spectrophotometer to determine the bacterial number in the solution.

### 2.3 Cell culture and generation of bone marrow-derived macrophages

BMDMs were generated and cultured as described previously (24). Briefly, bone marrow cells from ZBP1 KO and C57BL/6 mice were harvested from the tibias and femurs were differentiated into macrophages using DMEM (Gibco/Thermo Fisher Scientific, Waltham, MA) supplemented with 10% fetal bovine serum (FBS) (Life Technologies, Carlsbad, CA), 20% L929-cell conditioned medium (LCCM), 1% HEPES (Life Technologies) and 100 U/ml penicillin-streptomycin (Life Technologies), at 37°C in 5% CO_2_. At day 4 of culture, 10 mL of fresh medium was added. At day 7, cells completely differentiated into macrophages were detached and seeded in 24-well plates at a density of 5 x 10^5^ cells/well for use in experiments.

### 2.4 Macrophage stimulation with *Brucella abortus* or transfected DNA

Cultured macrophages from ZBP1 KO and C57BL/6 mice were infected *in vitro* with *B*. *abortus* at the multiplicity of infection (MOI) of 100:1 in DMEM with 1% FBS for the indicated times at 37°C in 5% CO_2_. *B. abortus* DNA was purified using the Illustra bacteria genomic Prep Mini Spin Kit (GE Healthcare, Buckinghamshire, United Kingdom) according to the manufacturer’s instructions. Then, the purified bacterial DNA was transfected (1 μg/mL) using FuGENE HD (Promega, Madison, WI) accordingly to manufacturer instructions. Culture supernatants and cell lysates were harvested and stored at -80°C until use.

### 2.5 STING knockdown in macrophages via small interfering RNA

Macrophages from ZBP1 KO and C57BL/6 mice were transfected with siRNA from siGENOME SMARTpools (Dharmacon, Lafayette, CO) using the GenMute siRNA transfection reagent according to the manufacturer’s instructions (SignaGen, Rockville, MD). siGENOME SMARTpool siRNA specific for mouse STING (M-055528-01) and a control siRNA pool were used (D-001206-14-05). Forty-eight hours after transfection, culture medium was replaced and macrophages were infected as described above.

### 2.6 Quantitative real-time PCR

Macrophages from ZBP1 KO and C57BL/6 mice treated as described above were homogenized in TRIzol reagent (Invitrogen, Carlsbad, CA, USA) to obtain total RNA accordingly to manufacturer guidelines. Then, RNA was treated with DNase I (Invitrogen) to remove genomic DNA followed by reverse transcription of 1 μg of total RNA using Illustra Ready-To-Go RT-PCR Beads (GE Healthcare, Chicago, IL) according to the manufacturer’s instructions. Real-time RT-PCR was performed using SYBR Green PCR master mix (Applied Biosystems, Foster City, CA) on a QuantStudio3 real-time PCR instrument (Applied Biosystems), using the following cycling parameters: 60°C for 10 min, 95°C for 10 min, 40 cycles of 95°C for 15 sec, and 60°C for 1 min, and a dissociation stage of 95°C for 15 sec, 60°C for 1 min, 95°C for 15 sec, and 60°C for 15 sec. The appropriate primers were used to amplify a specific fragment corresponding to specific gene targets as follows: BiP F: 5’-AGGATGCGGACATTGAAGAC-3’, R: 5’-AGGTGAAGATTCCAATTACATTCG-3’; XBP1(s) F: 5’-GAGTCCGCAGCAGGTG-3’, R: 5’-GTGTCAGAGTCCATGGGA-3’; IFN-β F: 5’-GCCTTTGCCATCCAAGAGATGC-3’, R: 5’-ACACTGTCTGCTGGTGGAGTTC-3’; IFN-α4 F: 5’-CCTGTGTGATGCAGGAACC-3’, R: 5’-TCACCTCCCAGGCACAGA-3’; β-actin F: 5’-GGCTGTATTCCCCTCCATCG-3’, R: 5’-CCAGTTGGTAACAATGCCATGT-3’. All data are presented as relative expression after normalization to the *β-actin* gene.

### 2.7 Cytokine measurements, LDH release determination and nitric oxide assay

Macrophage supernatants from ZBP1 KO and C57BL/6 mice were harvested from treated cells for cytokine, NO and lactate dehydrogenase (LDH) measurements. The murine cytokines (IL-1β, IL-6, IL-12 and TNF-α) were detected using ELISA kits (R&D systems, Minneapolis, MN), according to the manufacturer’s instructions. To evaluate NO production, the concentration of nitrite (NO_2_^−^) was assessed using the Griess reagent method as previously described (25). The LDH activity was measured using the CytoTox96 LDH release kit (Promega, Madison, WI), according to the manufacturer’s instructions.

### 2.8 Western blot analysis

Supernatants from treated macrophages were harvested and cells were lysed with M-PER Mammalian Protein Extraction Reagent (Thermo Fisher Scientific) supplemented with 1:100 protease inhibitor mixture (Sigma-Aldrich, St. Louis, MO). Then, equal volume of supernatants or equivalent amounts of protein of cell lysates were loaded onto 12% SDS-polyacrylamide gels. Following electrophoresis, bands were transferred to nitrocellulose membranes (Amersham Biosciences, Uppsala, Sweden) according to standard techniques. Membranes were blocked in Tris-buffered saline (TBS) with 0.1% Tween-20 containing 5% nonfat dry milk for 1 hr and then incubated at 4°C overnight with primary antibodies (IL-1β, clone 3A6; IRF-1, clone D5E4; BiP, clone C50B12; β-actin, clone 13E5; Cell Signaling Technology, MA, Danvers) (ZBP1, clone Zippy-1; p20 subunit of caspase-1, clone Casper-1; Adipogen, San Diego, CA). The membranes were washed three times for 5 min in TBS with 0.1% Tween 20 and incubated for 1 hr at room temperature with the appropriate HRP-conjugated secondary antibody (Cell Signaling Technology). Proteins were visualized using Luminol chemiluminescent HRP substrate (Millipore, Burlington, MA) in an Amersham Imager 600 (GE Healthcare). Densitometry analysis was performed using ImageQuant TL Software (GE Healthcare) and band intensities were normalized to β-actin. Data were relativized to the level of WT macrophages infected with *B. abortus* for 8 h assigned arbitrarily with the value of 1.0.

### 2.9 Measurement of *B. abortus* CFU in infected mice and macrophages

ZBP1 KO and C57BL/6 mice were infected *i.p.* with 1 x 10^6^ colony formation units (CFU) of *B. abortus* in 0.1 ml of saline (NaCl 0.9%). After 2 and 4 weeks post-infection, mice were sacrificed and spleens were used to determine the number of bacteria by CFU counting. For the measurement of viable intracellular bacteria *in vitro*, infected macrophages were washed twice with PBS and then lysed for 10 min at room temperature in 1 mL of PBS containing 0.1% Triton X-100 under manual agitation. To assess *B. abortus* CFU, spleens and cells lysates were serially diluted in saline and plated in duplicate on BB agar. Plates were incubated for 3 days at 37°C and CFU number was determined.

### 2.10 Proinflammatory cytokine production in Brucella‐primed spleen cells

Spleen cells were harvested from infected mice and treated with an ammonium-chloride-potassium buffer (0.15 M NH_4_Cl, 1.0 mM KHCO_3_, 0.1 mM ethylenediaminetetraacetic acid [EDTA]; pH 7.2) to lyse red blood cells. After washing, the cells were resuspended in RPMI medium (Life Technologies) supplemented with 2 mM L-glutamine (Life Technologies), 25 mM HEPES, 10% heat-inactivated FBS, 100 U/mL penicillin G sodium, and 100 mg/mL streptomycin sulfate, and adjusted to 1 × 10^6^ cells per well in a 96-well plate. Splenocytes were stimulated with *B. abortus* (MOI of 100:1), 1 µg/ml *Escherichia coli* LPS (Sigma-Aldrich), or 5 µg/ml Concanavalin A (ConA) (Sigma-Aldrich). Spleen cells were incubated at 37°C in 5% CO_2_, and culture supernatants were collected 48 or 72 hours after stimulation to measure TNF-α or IFN-γ, respectively, by ELISA (R&D Systems).

### 2.11 Statistical analysis

Data analysis and graphing were performed using GraphPad Prism 5 software (GraphPad Software, San Diego, CA). All quantitative data are expressed as mean ± standard deviation. The data presented are representative of three independent experiments. A p value less than 0.05 (p<0.05) was considered statistically significant using two-way ANOVA test.

## 3 Results

### 3.1 ZBP1 is involved in type I interferon expression

**Figure 1:**
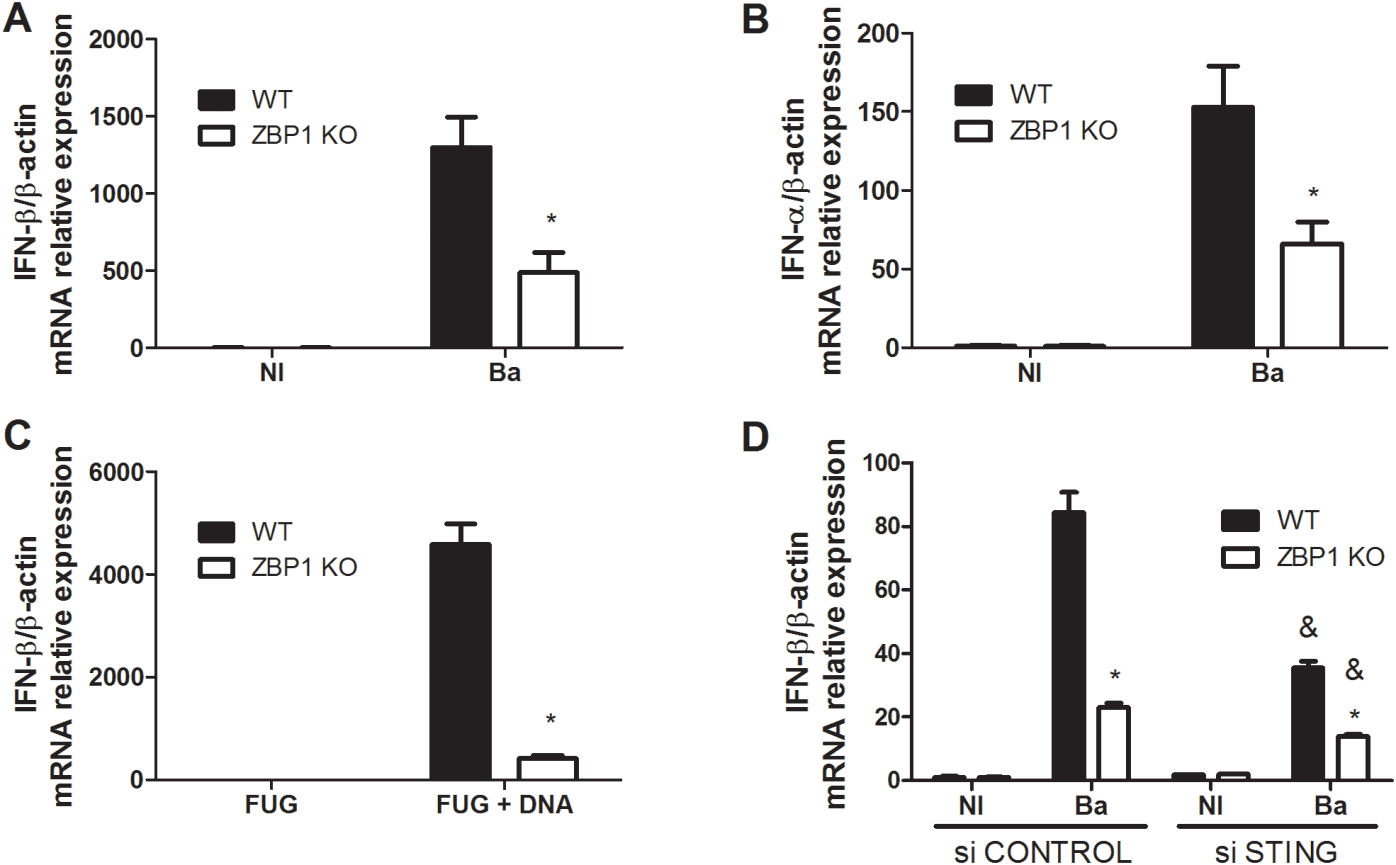
*B*. *abortus* induces ZBP1 activation and type I interferon expression. Macrophages from wild-type (WT) or ZBP1 KO mice were infected with *B*. *abortus* (Ba) for 16 h and the *IFN-β* **(A)** and *IFN-α* **(B)** expression levels were determined by real-time RT-PCR. Non-infected cells (NI, control) were incubated under the same experimental conditions without bacteria. **(C)** Macrophages from wild-type (WT) or ZBP1 KO mice were stimulated with transfected *B*. *abortus* DNA for 16 h and the *IFN-β* expression levels were determined by real-time RT-PCR. Fugene alone (FUG) was used as control. **(D)** Macrophages from wild-type (WT) or ZBP1 KO mice were transfected with non specific siRNA (si CONTROL) or STING siRNA (si STING) for 2 days. Then, cells were infected with *B*. *abortus* for 16 h and the *IFN-β* expression levels were determined by real-time RT-PCR. The data **(A-D)** are presented as mean ± SD. **(A-C)**, * (comparison between WT and KO), p < 0.05, two-way ANOVA. **(D)**, * (comparison between WT and KO) or & (comparison between si CONTROL-treated and si STING-treated), p < 0.05, two-way ANOVA.

In the context of intracellular *Brucella* infection, the activation of innate immune sensors can occur through distinct mechanisms leading to type I IFN response (21). To evaluate the impact of ZBP1 in this pathway, BMDMs were obtained from both ZBP1 KO and C57BL/6 mice. Subsequently, these cells were exposed to the virulent *B. abortus* S2308 strain, and the expression of the *IFN-β* gene (**Figure 1A**) and *IFN-α* gene (**Figure 1B**) was assessed. The results revealed a significant reduction in the expression of both type I IFN genes in ZBP1 KO macrophages compared to WT cells. In prior studies, the involvement of ZBP1 in DNA-mediated innate immune responses was recognized, and ZBP1 was designated as a cytosolic DNA sensor (3). Therefore, we examined the influence of transfected *Brucella abortus*-derived DNA on *IFN-β* expression through ZBP1 activation, using macrophages from ZBP1 KO and WT mice. As observed in **Figure 1C**, *IFN-β* expression induced by transfected DNA was dependent on the presence of ZBP1. Taken together, our data demonstrated that both *Brucella* infection and bacterial DNA transfection induces type I IFN responses in a ZBP1-dependent manner.

Moreover, we addressed the cooperation between the STING and ZBP1 in driving type I IFN expression during *B. abortus* infection. Hence, we performed siRNA silencing of STING in ZBP1 KO and WT macrophages. The knockdown of siRNA led to decreased expression of *IFN-β* in both ZBP1 KO and WT cells compared to cells treated with the control (scramble siRNA) (**Figure 1D**). The data suggest that both STING and ZBP1 contribute to type I IFN responses induced by *Brucella*-infected macrophages.

### 3.2 ZBP1 enhances the activation of the unfolded protein response upon *B. abortus* infection

**Figure 2:**
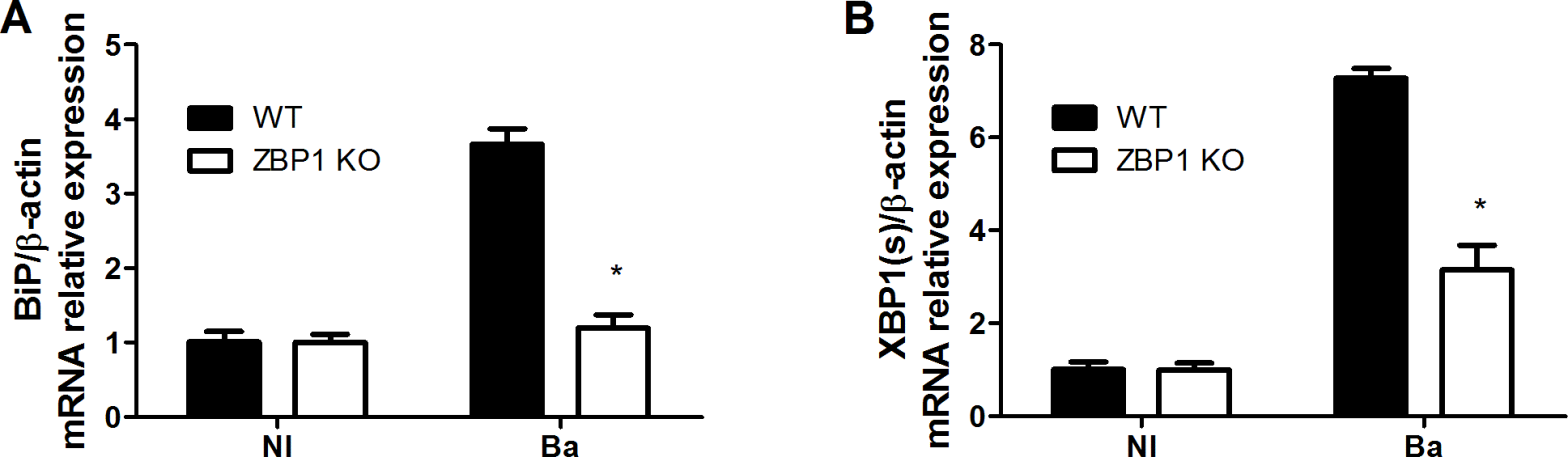
ZBP1 promotes the *Brucella*-induced UPR response. Macrophages from wild-type (WT) or ZBP1 KO mice were infected with *B*. *abortus* (Ba) for 16 h and the BiP **(A)** and XBP1(s) **(B)** expression levels were determined by real-time RT-PCR. Non-infected cells (NI, control) were incubated under the same experimental conditions without bacteria. The data **(A, B)** are presented as mean ± SD. **(A, B)**, * (comparison between WT and KO), p < 0.05, two-way ANOVA.

Previous data unveiled that *B. abortus* infection led to UPR induction and enhanced expression of the UPR downstream targets, BiP and XBP1(s). Moreover, it was shown the crucial role of IFN-β in triggering UPR during *B. abortus* infection (22). Given that ZBP1 plays a pivotal role in type I IFN response, we examined the involvement of this receptor in the UPR activation during *B. abortus* infection. In this regard, macrophages from ZBP1 KO and WT mice were infected to assess the expression of *BiP* (**Figure 2A**) and *XBP1*(s) (**Figure 2B**). The results indicated increased expression of both *BiP* and *XBP1*(s) in a ZBP1-dependent manner. Therefore, our data suggest that ZBP1 plays a role in controlling the UPR activation upon *B. abortus* infection.

### 3.3 ZBP1 contributes to type I interferon signaling during *B. abortus* infection

**Figure 3:**
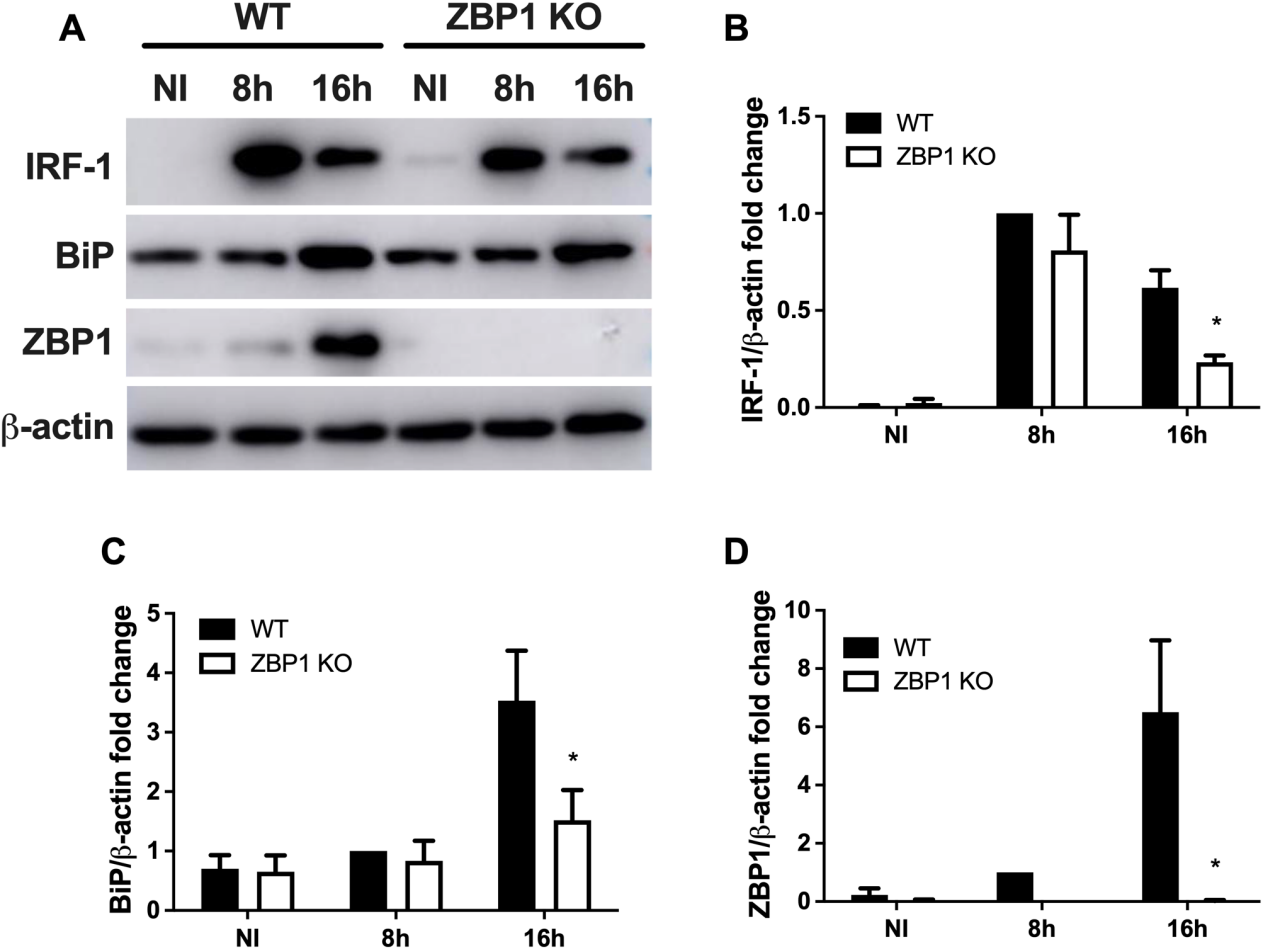
ZBP1 participates in the type I interferon signaling elicited by *Brucella*. **(A)** Western blot analysis of IRF-1, BiP and ZBP1 in wild-type (WT) or ZBP1 KO macrophages lysates, non-infected (NI) or infected with *B*. *abortus* at 8 h or 16 h Equal loading was verified by measuring β-actin levels in the corresponding cell lysates. The densitometry analysis of Western blot of IRF-1 **(B)**, BiP **(C)** and ZBP1 **(D)** were performed relative to β-actin. The data **(B-D)** are presented as mean ± SD. **(B-D)** * (comparison between WT and KO), p < 0.05, two-way ANOVA.

IRF-1 operates as a transcriptional regulator, activating the expression of genes associated with protection against invading pathogens (26). IRF-1 functions downstream of IFN expression, participating in the signal transduction pathway initiated by IFN during infection (27). Regarding *B. abortus* infection, it was previously demonstrated that the expression of IRF-1 depends on the activation of IFNAR by IFN-β (21). Given this context, we evaluated the level of IRF-1 protein expression in macrophages derived from WT and ZBP1 KO mice. We noted a significant increase in IRF-1 protein level in WT cells following bacterial infection compared to the non-infected cells (**Figures 3A, B**). Moreover, this upregulation was partially dependent on ZBP1, as KO macrophages exhibited reduced IRF-1 protein level 16 hours post-infection. Furthermore, we assessed the protein level of BiP upon *B. abortus* infection (**Figures 3A, C**). It was observed that the increase in BiP protein level occurred at 16 hours post-infection, and this enhancement relies in the presence of ZBP1. This result strengthens the conception that ZBP1 contributes to activate the UPR. Finally, we examined the level of ZBP1 protein stimulated by the infection (**Figures 3A, D**). The results revealed a significant increase in ZBP1 protein level only at 16 hours post-infection. Collectively, our data indicates that ZBP1 stimulated by *B. abortus* infection drives IFN-β expression potentially contributing to IRF-1 expression.

### 3.4 ZBP1 is dispensable for production of proinflammatory mediators during *B. abortus* infection

**Figure 4:**
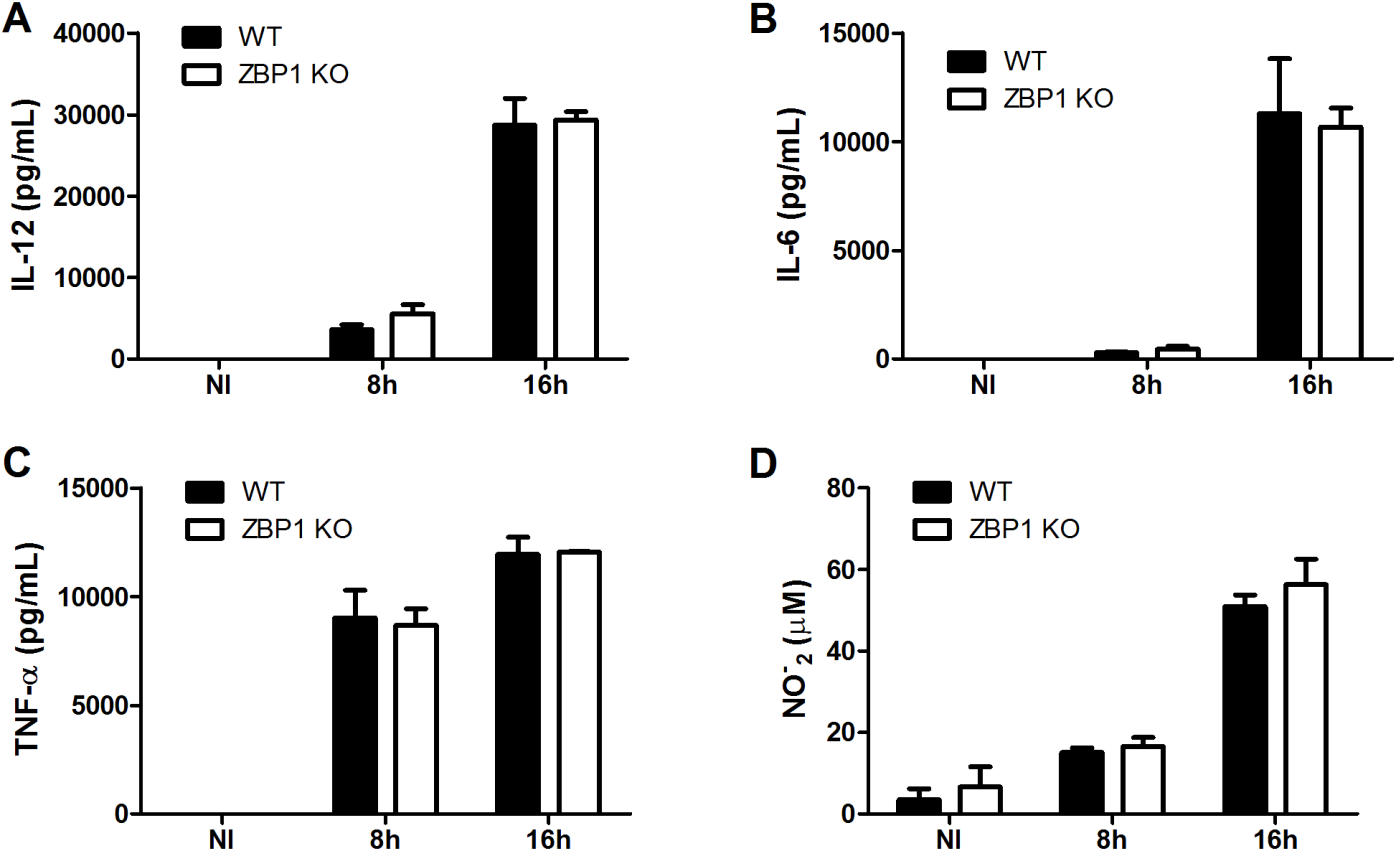
ZBP1 is not necessary for the production of proinflammatory cytokines and NO during *B. abortus* infection. The cytokines IL-12 **(A)**, IL-6 **(B)** and TNF-α **(C)** produced by wild-type (WT) or ZBP1 KO macrophages, non-infected (NI) or infected with *B. abortus* at 8 h or 16 h, were detected in cell supernatants using ELISA. **(D)** NO_2_^−^ (nitrite) accumulation in cell supernatants from wild-type (WT) or ZBP1 KO macrophages, non-infected (NI) or infected with *B*. *abortus* at 8 h or 16 h, were measured by Griess reaction. The data **(A-D)** are presented as mean ± SD. No statistical difference was observed (comparison between WT and KO), p < 0.05, two-way ANOVA.

In addition to the type-I IFN response during ZBP-1 activation, the NF-κB signaling pathway leading to proinflammatory cytokine production also constitutes a line of defense against pathogenic infections (7). In that context, ZBP1 also emerged as a regulator of proinflammatory cytokine production, such as IL-6 and TNF-α (11). Thus, we aimed to evaluate the participation of ZBP1 in the secretion of proinflammatory cytokines by macrophages during *B. abortus* infection. Macrophages derived from ZBP1 KO mice exhibited similar levels of IL-12, IL-6, and TNF-α in comparison to WT infected macrophages at all time points tested (**Figures 4A–C**). Moreover, considering nitric oxide (NO) as another classical marker of the inflammatory macrophage profile, we assessed NO production in infected cells. The findings revealed that ZBP1 KO infected macrophages displayed no difference in NO production compared to infected cells from WT mice (**Figure 4D**). Taken together, the data suggest that the production of proinflammatory cytokines and NO by macrophages infected with *B. abortus* occurs independently of ZBP1 activation.

### 3.5 Inflammasome activation and cell death during *B. abortus* infection are ZBP1-independent

**Figure 5:**
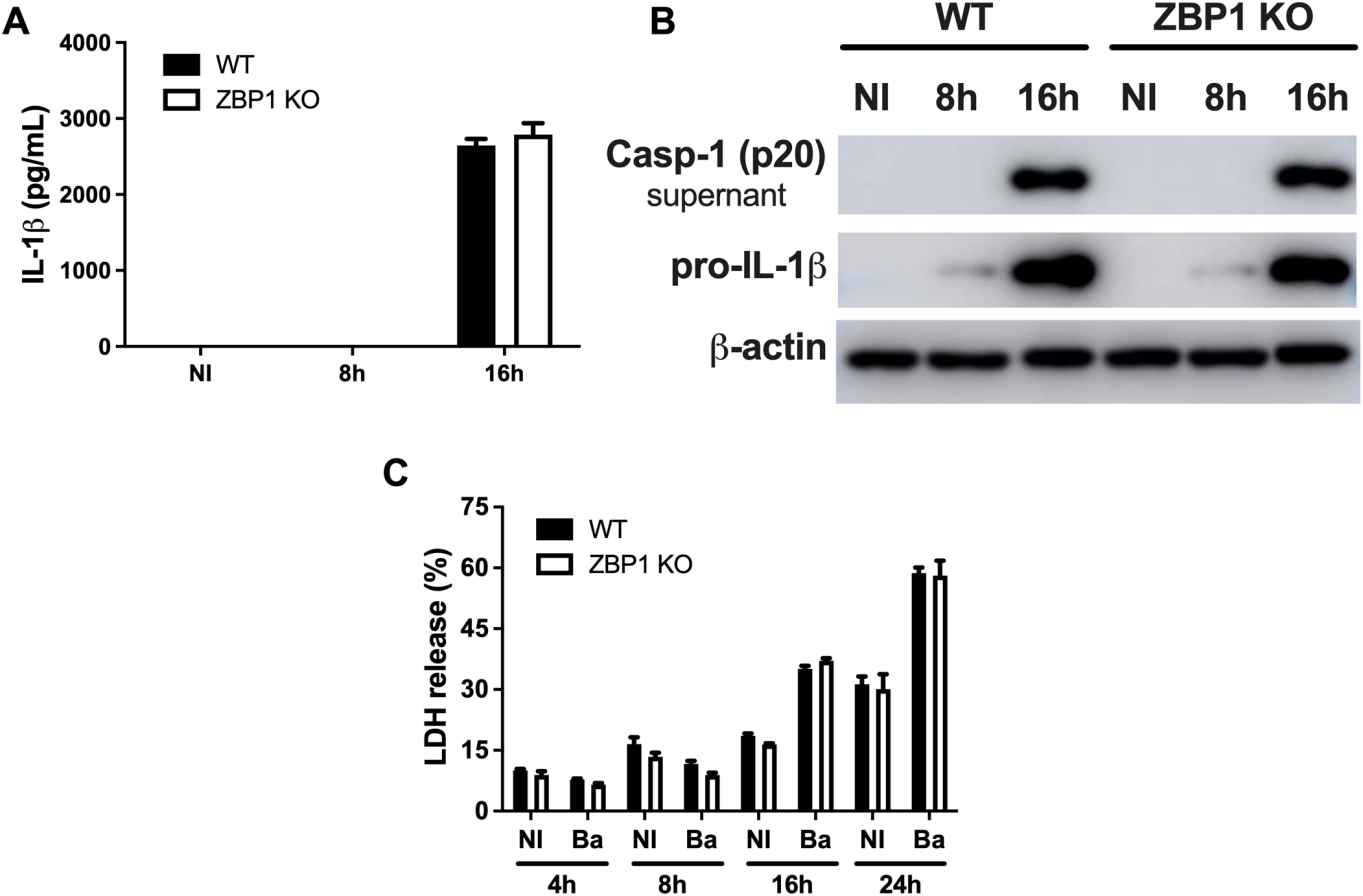
ZBP1 is dispensable for inflammasome activation and cell death during *B. abortus* infection. **(A)** The cytokine IL-1β released by wild-type (WT) or ZBP1 KO macrophages, non-infected (NI) or infected with *B*. *abortus* at 8 h or 16 h, were detected in cell supernatants using ELISA. **(B)** Western blot analysis of wild-type (WT) or ZBP1 KO macrophages, non-infected (NI) or infected with *B. abortus* at 8 h or 16 h The protein pro-IL-1β was detected in cell lysates, and the active form of caspase-1 (p20 subunit) in supernatants. Equal loading was verified by measuring β-actin levels in the corresponding cell lysates. **(C)** Cell death was assessed by measuring LDH release in the supernatant of wild-type (WT) or ZBP1 KO macrophages, non-infected (NI) or infected with *B. abortus* (Ba) at the indicated time points. The data **(A, C)** are presented as mean ± SD. No statistical difference was observed (comparison between WT and KO), p < 0.05, two-way ANOVA.

A growing body of evidence showed that ZBP1 induces inflammasome activation and subsequent IL-1β secretion (28–30). Thus, to gain insight into the role of ZBP1 in promoting inflammasome activation in response against *B. abortus* infection, we assessed the production of IL-1β and caspase-1 processing in macrophages. The data showed no difference in the secretion of IL-1β when comparing macrophages derived from ZBP1 KO mice to those from WT mice (**Figure 5A**). Furthermore, both WT and ZBP1 KO infected macrophages exhibited equivalent levels of pro-IL-1β (**Figure 5B**), and no difference was observed regarding the presence of caspase-1 p20 subunit in cell supernatants (**Figure 5B**), suggesting no influence of ZBP1 in inflammasome assembly and activation.

Cell death is closely interconnected to the host immune response during infection, and ZBP1 was previously linked to cell death measured by lactate dehydrogenase (LDH) release (28). In addition, we demonstrated previously that *B. abortus* infection induces pyroptosis and LDH release in a process dependent on caspase-11 activation and gasdermin-D cleavage (31). Therefore, we assessed the involvement of ZBP1 in macrophage cell death mediated by bacterial infection through LDH release in a time-lapse experiment. It was observed that LDH release is prominent after 16 hours of infection compared to non-infected cells. Furthermore, there is no difference concerning cell death between WT and ZBP1 KO macrophages at any assessed time point (**Figure 5C**). Thus, the data indicates that ZBP1 is not essential for the cell death induced by *B. abortus* in infected macrophages.

### 3.6 ZBP1 does not contribute to control of *B. abortus* infection

**Figure 6:**
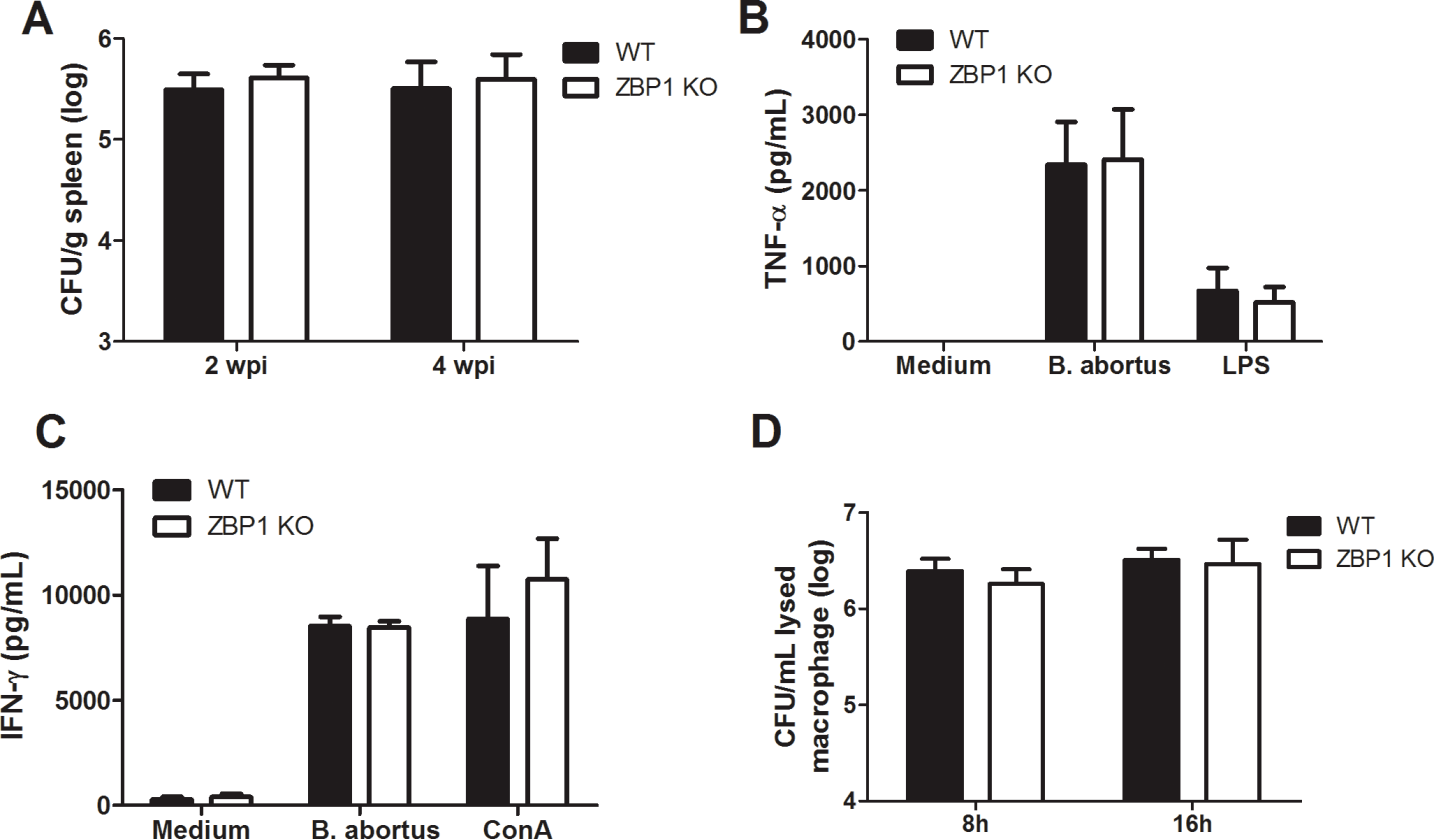
ZBP1 is not required for the control of *B*. *abortus* infection. **(A)** Residual *B*. *abortus* CFU in the spleen of wild-type (WT) or ZBP1 KO mice infected intraperitoneally with *B*. *abortus* were determined at 2 and 4 weeks post-infection (wpi). Splenocytes from 4-week-infected mice were stimulated with *B*. *abortus*, 5 μg/ml ConA, 1 μg/ml LPS, or medium as a negative control. Supernatants from the splenocytes were harvested 48 or 72 hours after stimulation and analyzed by ELISA for TNF-α **(B)** or IFN-γ **(C)**, respectively. **(D)** Macrophages from wild-type (WT) or ZBP1 KO mice were infected with *B*. *abortus* for 8 h or 16 h and the CFU assessed in cell lysates. The data **(A-D)** are presented as mean ± SD. No statistical difference was observed (comparison between WT and KO), p < 0.05, two-way ANOVA.

Previously, our group demonstrated that mice deficient in the IFN-αβ receptor controlled *Brucella* infection more efficiently than wild-type animals (32). This data indicated the detrimental role of type I IFN signaling to the host during *B. abortus* infection. Since *IFN-β* expression is partially diminished in ZBP1 KO mice, we explored the role of ZBP1 in host defense against *B. abortus*. Thus, we infected both WT and ZBP1 KO mice and then assessed the bacterial load in the spleen at 2 and 4 weeks post-infection (wpi) (**Figure 6A**). As observed, there is no difference between WT and ZBP1 KO mice concerning CFU counts in spleen at both analyzed time points post-infection. In addition, to further explore the role of ZBP-1 *in vivo*, we analyzed the production of proinflammatory cytokines during *B. abortus* infection. Splenocytes from wild-type and ZBP1 KO infected mice were stimulated with live bacteria, ConA, or LPS as controls, and cytokine secretion was determined. Analysis of all stimuli demonstrated that ZBP1 KO-infected mice produced similar levels of the proinflammatory cytokines TNF-α (**Figure 6B**) and IFN-γ (**Figure 6C**) compared to WT infected animals.

Furthermore, we evaluated the CFU counts following 8 hours and 16 hours of infection in macrophages derived from ZBP1 and WT mice (**Figure 6D**). As observed, there is no difference in bacterial replication within macrophages at any of the analyzed time points. Therefore, these data suggest that ZBP1 is not essential for controlling either *Brucella* infection *in vivo* or bacterial replication inside macrophages.

## 4 Discussion

Innate immune cells are often confronted by pathogenic bacteria that are able to survive and replicate intracellularly. The recognition of pathogen-associated molecular patterns (PAMPs) by pattern recognition receptors (PRRs) is pivotal for initiating a proper immune response leading to activation of potent antimicrobial effector pathways against bacterial infection (33). Regarding *B. abortus*, DNA is considered a major bacterial PAMP which activates the host innate immune system involving TLR9, AIM2 and STING sensors (34). Here we demonstrated that ZBP1 elicit DNA-mediated innate immune responses by inducing type I IFN expression during *B. abortus* infection. Corroborating our data, it was previously demonstrated that ZBP1 interacts with synthetic B-form DNA, and longer DNA sequences were found to be more effective in inducing IFN-β production compared to shorter sequences (4). In addition, it becomes evident that the cytosolic DNA-sensing system is redundant, as suppression of ZBP1 expression only partially inhibits *IFN-β* expression (4). Regarding *B. abortus* infection, our present study indicates that both STING and ZBP1 contribute to type I IFN signaling pathway. This diversity of pathways underscores the adaptability of the immune system, which may compensate for the absence of one single receptor during bacterial infection.

The accumulation of misfolded or unfolded proteins in the ER activates the UPR. This pathway is crucial for maintaining cellular homeostasis and promoting cell survival under conditions of ER stress (35). *Brucella* is known to traffic to the ER and activates the UPR (36). Activation of the UPR elicited by *B. abortus* infection is dependent on STING and leads to the upregulation of chaperone proteins such as BiP and the splicing of XBP1. Notably, IFN-β production and signaling participates in this UPR activation in response to *B. abortus* infection (22). In this context, we observed here that ZBP1 is implicated in the activation of the UPR pathway during *B. abortus* infection. This was evidenced by impaired upregulation of BiP and spliced XBP1 in ZBP1 KO macrophages compared to WT cells. These findings suggest that ZBP1 is involved in coordinating the UPR pathway in response to *B. abortus*, highlighting its role in cellular stress response during bacterial infection. These findings also suggest that the type I IFN production elicited by ZBP1 activation is correlated with the UPR, similar to the role observed with STING. However, further investigations are needed to elucidate novel specific mechanisms by which ZBP1 regulates the UPR activation during *B. abortus* infection. In addition, UPR pathway was previously associated to establish a safe replication zone in ER favoring *Brucella* replication (22, 37, 38). However, our data show no evidence of ZBP1 participating in the control of *B. abortus* replication inside macrophages. Therefore, it seems possible that partial inhibition of UPR sensors by ZBP1 is not sufficient to alter *Brucella* replication.

The production of proinflammatory cytokines is associated with the immune response against *B. abortus* infection (39). Regarding ZBP1, it was previously shown that this sensor activates NF-κB signaling eliciting proinflammatory cytokines production in response to cytosolic DNA (3, 40). The role of ZBP1 in proinflammatory cytokine and NO production during *B. abortus* infection appears to be limited based on our findings. The results presented here suggest that *B. abortus* induces proinflammatory mediators by activating other PRRs, and ZBP1 is dispensable in this process. It is well known that several factors contribute to the induction of immune response against *B. abortus* infection (18). Notably, the recognition of *Brucella*-derived molecules by various innate immune receptors leads to the activation of signaling pathways that culminate in proinflammatory cytokine production (41). For instance, cytosolic *Brucella* DNA recognition by STING pathway also triggered proinflammatory cytokine production, and a STING-dependent resistance to *B. abortus* infection was described (21). In this regard, STING activation upon *B. abortus* infection also induced the M1-type macrophages (classically activated macrophages) and NO production, which are associated with host protection (24). The data provide here demonstrated that ZBP1 is not required for the control of *B. abortus* infection.

When triggered by pathogen infection, ZBP1 potentially initiates PANoptosis which involves the activation of cell death pathways such as pyroptosis, apoptosis, and necroptosis (6). ZBP1-mediated PANoptosis was characterized by NLRP3 inflammasome activation with LDH and IL-1β release during viral infection (11). In our previous study on *B. abortus* infection, we demonstrated the involvement of NLRP3 and AIM2 in inflammasome activation (20). Therefore, we aimed to evaluate the participation of ZBP1 in this process. The data presented here reveal that ZBP1 is not required for IL-1β release, caspase-1 processing, and cell death induced by *B. abortus* infection, suggesting a mechanism of NLRP3/AIM2 activation that is independent of ZBP1. These findings differ from the response to other bacterial pathogens mediated through ZBP1 activation. For instance, ZBP1 cooperates with pyrin to form a complex that drives AIM2-mediated caspase-1 activation and cell death, contributing to host defense against *Francisella novicida* (12). In addition, ZBP1 contributes to cell death induction during Mtb infection, playing a key role in necroptosis by promoting MLKL phosphorylation (13).

In summary, we proposed that ZBP1 activation contributes to the production of type I IFN in response to *B. abortus* infection or its derived DNA. Moreover, ZBP1 participates in the activation of the UPR pathway influencing the expression of BiP and XBP1(s). However, ZBP1 is dispensable for controlling *B. abortus* replication within macrophages or infected mice. ZBP1 also does not significantly impact proinflammatory cytokine secretion or inflammasome activation. These findings highlight ZBP1 as a key player in type I IFN production and UPR activation in response to *B. abortus*, suggesting a specific role for ZBP1 in the innate immune response against this pathogen. Our results shown here provide insights into the interplay between ZBP1-mediated innate immunity and cellular stress responses, contributing to our understanding of host-pathogen interactions.

## Data Availability

The raw data supporting the conclusions of this article will be made available by the authors, without undue reservation.
