# Supplementary material for: ZBP1 senses Brucella abortus DNA triggering type I interferon signaling pathway and unfolded protein response activation
Source: Front Immunol. 2025 Jan 9;15:1511949. doi: 10.3389/fimmu.2024.1511949 (PMC11754416; doi:10.3389/fimmu.2024.1511949)
Supplement: Supplementary file 1 [file DataSheet1.pdf]

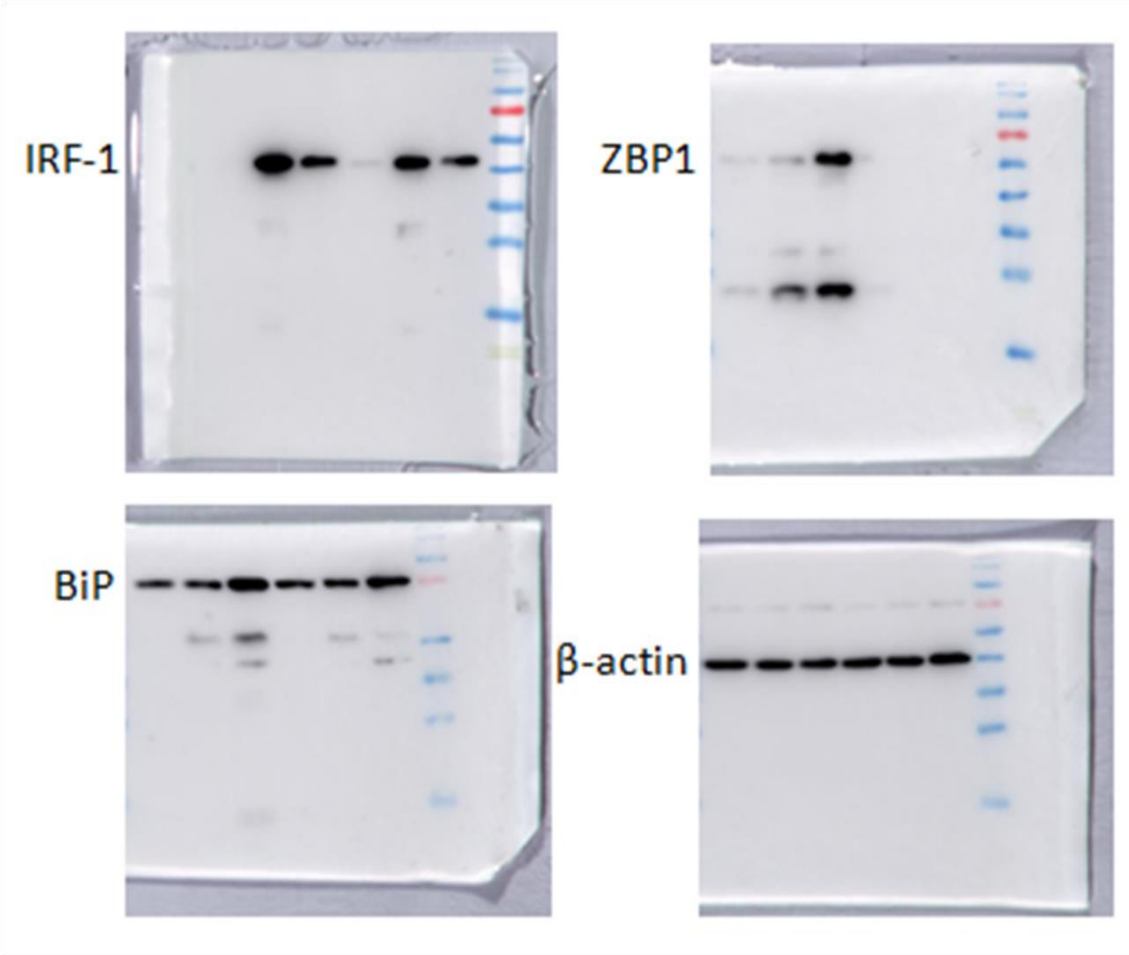

Supplementary Figure 1. Original Western blot membranes (referencing Figure 3) from immunodetection using IRF-1, BiP, ZBP1, and β-actin antibodies.

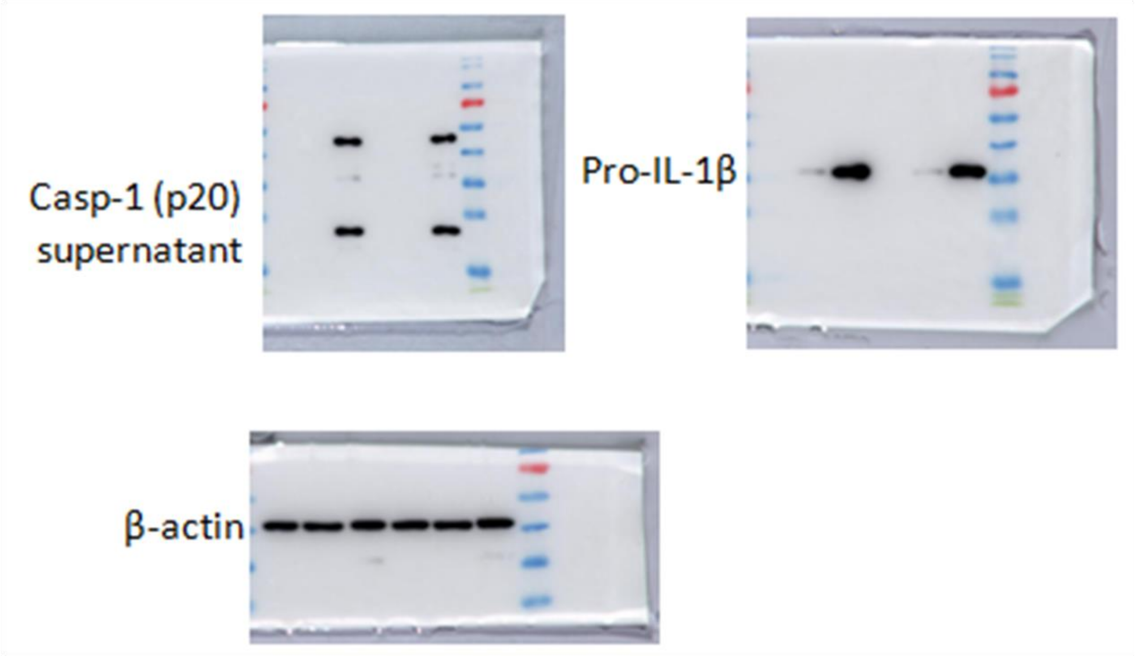

Supplementary Figure 2. Original Western blot membranes (referencing Figure 5) from immunodetection using caspase-1, IL-1β and β-actin antibodies.
